# Association between Sleep Duration and Chronic Rhinosinusitis among the Korean General Adult Population: Korea National Health and Nutrition Examination Survey

**DOI:** 10.1038/s41598-019-43585-w

**Published:** 2019-05-09

**Authors:** Jeong-Whun Kim, Hahn Jin Jung, Hyo Geun Choi, Chae-Seo Rhee, Jee Hye Wee

**Affiliations:** 1 Department of Otorhinolaryngology-Head and Neck Surgery, Seoul National University Bundang Hospital, Seoul National University College of Medicine, Seongnam, South Korea; 2 Department of Otorhinolaryngology-Head and Neck Surgery, Chungbuk National University Hospital, Chungbuk National University College of Medicine, Cheongju, South Korea; 3 Department of Otorhinolaryngology-Head and Neck Surgery, Hallym University Sacred Heart Hospital, Hallym University College of Medicine, Anyang, South Korea

**Keywords:** Health care, Epidemiology

## Abstract

An association between sleep duration and a wide spectrum of diseases has been reported, but little is known about its relationship with chronic rhinosinusitis (CRS). The present study aimed to investigate whether sleep duration was associated with CRS after adjusting for potential confounding factors in Korean adults. We analyzed data from the Korea National Health and Nutrition Examination Survey 2005–2009 and enrolled 24,658 participants aged ≥20 years. Data regarding sociodemographic characteristics, self-reported sleep duration, CRS, and other medical diseases were collected from questionnaires. Multiple logistic regression analyses were used to identify the relationship between sleep duration and CRS. The overall prevalence of CRS was 4.4%. Subjects with sleep duration ≤5 hours showed a highest prevalence for CRS (6.1%), compared to subjects with longer sleep duration (*p* = 0.02). After adjusting for covariates (age, sex, household income, residency, dwelling type, education, depression, alcohol, allergic rhinitis, chronic otitis media, angina or myocardial infarction, asthma, chronic obstructive pulmonary disease, bronchiectasis, and gastric ulcer), the sleep duration of ≤5 hours was significantly associated with CRS (OR = 1.502; 95% CI = 1.164–1.938). Only in older subgroup (≥50 years old), shorter sleep duration (≤5 hours) showed higher odds for CRS. These results suggested that sleep duration may be negatively associated with CRS in older Korean adults.

## Introduction

Chronic rhinosinusitis (CRS) is a common chronic respiratory disease, affecting 6.9–27.1% of the European population according to recent epidemiological studies^1^ conducted under the European Position Paper on Rhinosinusitis and Nasal Polyps (EPOS) 2012 guidelines^2^. Data from the Korea National Health and Nutrition Examination Survey (KNHANES) 2005 indicate a 6.95% prevalence of CRS in Korea. In addition, CRS has been recognized as a socioeconomic burden that negatively affects quality of life^3,4^.

Several epidemiological studies have reported that sleep duration is associated with a wide spectrum of diseases^5–10^. This was particularly evident for rhinologic diseases, wherein an association between reduced self-reported sleep duration and allergic rhinitis (AR) was reported^11^. However, little is known about the relationship between sleep duration and CRS.

For CRS, previous studies have demonstrated that patients with CRS have reduced sleep quality and poor quality of life^12^. In addition, improved sleep outcomes have been reported after endoscopic sinus surgery^13^. However, these studies did not focus on sleep duration, but rather on sleep quality or sleep dysfunction such as obstructive sleep apnea. Furthermore, the association between sleep duration and CRS has not been analyzed in the general population.

The aim of this study was to investigate whether self-reported sleep duration was associated with CRS after adjusting for potential confounding factors in the Korean general adult population.

## Results

### Characteristics of participants

The mean age of the study population was 44.5 ± 0.2 (range, 20–99) years, and the mean daily sleep duration was 6.85 ± 0.01 h. Table 1 shows the clinical characteristics of the study population. Subjects with CRS were younger and had a higher education level than subjects without CRS. Participants with CRS indicated that they were under more depressive symptoms and drinking than those without CRS. All of AR, chronic otitis media, angina or myocardial infarction, asthma, chronic obstructive pulmonary disease, bronchiectasis, gastric ulcer, and sleep duration were significantly associated with CRS.

**Table 1:** Clinical characteristics of the Korea National Health and Nutrition Examination Survey 2005–2009 participants aged ≥20 years old.

Variables	CRS (−), n = 23607	CRS (+), n = 1051	*p*-value
Age, ≥50 (%)	34.8 ± 0.5	30.8 ± 1.5	0.009
Sex, male (%)	49.6 ± 0.3	51.7 ± 1.7	0.239
Education, low (%)	35.9 ± 0.5	26.2 ± 1.5	<0.001
Household income, low (%)	41.9 ± 0.7	41.6 ± 2.0	0.860
Residency, urban (%)	46.9 ± 0.8	50.5 ± 2.0	0.051
Dwelling type, apartment (%)	51.7 ± 0.6	54.6 ± 1.8	0.120
Occupation, blue collar (%)	20.1 ± 0.5	20.3 ± 1.6	0.881
Stress (%)			0.156
None	15.1 ± 0.3	14.3 ± 1.4	
Low	55.1 ± 0.4	52.9 ± 1.9	
High	24.5 ± 0.3	25.6 ± 1.7	
Very High	5.3 ± 0.2	7.2 ± 1.0	
Suicidal ideation (%)	16.5 ± 0.3	19.1 ± 1.5	0.071
Depression (%)	14.0 ± 0.3	17.2 ± 1.4	0.017
Smoking (%)			0.439
Current smoker	44.9 ± 0.4	43.9 ± 2.1	
Ex-smoker	1.8 ± 0.1	1.4 ± 0.2	
Never smoker	53.3 ± 0.4	54.7 ± 2.1	
Alcohol, <1/month (%)	52.8 ± 0.6	45.1 ± 1.9	<0.001
Allergic rhinitis (%)	9.4 ± 0.2	22.4 ± 1.4	<0.001
Chronic otitis media (%)	4.2 ± 0.2	7.9 ± 1.0	<0.001
Hypertension (%)	13.8 ± 0.3	15.2 ± 1.2	0.231
Angina or Myocardial infarction (%)	1.4 ± 0.1	2.6 ± 0.5	0.005
Diabetes mellitus (%)	5.4 ± 0.2	4.4 ± 0.6	0.146
Hyperlipidemia (%)	4.9 ± 0.2	5.8 ± 0.8	0.217
Asthma (%)	2.6 ± 0.1	5.6 ± 0.8	<0.001
Chronic obstructive pulmonary disease (%)	0.7 ± 0.0	1.9 ± 0.4	<0.001
Bronchiectasis (%)	0.3 ± 0.0	0.9 ± 0.3	0.001
Gastric ulcer (%)	4.2 ± 0.1	6.5 ± 0.7	<0.001
Liver cirrhosis (%)	0.2 ± 0.0	0.1 ± 0.1	0.906
Atopic dermatitis (%)	4.6 ± 0.1	5.9 ± 0.8	0.059
Chronic renal failure (%)	0.3 ± 0.0	0.3 ± 0.2	0.900
Sleep duration (%)			0.020
≤5 hours	13.5 ± 0.3	17.5 ± 1.6	
6 hours	26.3 ± 0.4	27.1 ± 1.6	
7 hours	28.9 ± 0.1	28.0 ± 1.8	
8 hours	23.2 ± 0.4	19.3 ± 1.4	
≥9 hours	8.2 ± 0.2	8.1 ± 1.1	

Data are presented as the mean percentage ± SE. CRS: Chronic rhinosinusitis.

### Characteristics of participants according to sleep duration

Table 2 shows the prevalence of CRS of the participants and their characteristics according to sleep duration. The subjects were divided into 5 subgroups according to sleep duration: ≤5 hours, 6 hours, 7 hours, 8 hours and ≥9 hours. The subjects with sleep duration ≤5 hours showed the highest prevalence of CRS (6.1%), compared to subjects with longer sleep duration (*p* = 0.02). Age (*p* < 0.001), sex (*p* < 0.001), education level (*p* < 0.001), household income (*p* < 0.001), residency (*p* < 0.001), dwelling type (*p* < 0.001), occupation (*p* = 0.003), stress level (*p* < 0.001), suicidal ideation (*p* < 0.001), depression (*p* < 0.001), smoking (*p* = 0.004), alcohol income (*p* < 0.001), AR (*p* = 0.010), hypertension (*p* < 0.001), angina or myocardial infarction (*p* < 0.001), diabetes mellitus (*p* < 0.001), hyperlipidemia (*p* < 0.001), asthma (*p* < 0.001), gastric ulcer (*p* < 0.001), liver cirrhosis (*p* = 0.008), and chronic renal failure (*p* = 0.011) were significantly associated with sleep duration. Participants with sleep durations of ≤5 hours were more likely to be older, lower education level and household income, apartment residency, under high or very high stress level, less alcohol consumer, having no AR, and having hypertension, angina or myocardial infarction, diabetes mellitus, hyperlipidemia, asthma, gastric ulcer, and liver cirrhosis compared with any other sleep duration subgroup. Female, white collar occupation, and subjects who had suicidal ideation, depressive symptoms, and chronic renal failure exhibited a U-shaped distribution by sleep duration.

**Table 2:** Characteristics of participants across categories of sleep duration. Data are presented as the mean percentage ± SE.

Characteristics	≤5 h/day, n = 3723	6 h/day, n = 6295	7 h/day, n = 7032	8 h/day, n = 5594	≥9 h/day, n = 2014	*p*-value
Chronic rhinosinusitis (%)	6.1 ± 0.6	4.9 ± 0.3	4.6 ± 0.3	4.0 ± 0.3	4.7 ± 0.6	0.020
Age, ≥50 (%)	55.9 ± 1.2	33.4 ± 0.8	30.2 ± 0.8	31.0 ± 0.9	36.1 ± 1.5	<0.001
Sex, male (%)	43.7 ± 1.0	53.5 ± 0.8	50.6 ± 0.7	48.8 ± 0.8	42.6 ± 1.5	<0.001
Education, low (%)	50.2 ± 1.2	28.4 ± 0.8	25.4 ± 0.7	27.3 ± 0.9	36.3 ± 1.5	<0.001
Household income, low (%)	53.8 ± 1.4	38.8 ± 1.1	36.9 ± 1.0	41.6 ± 1.1	49.0 ± 1.7	<0.001
Residency, urban (%)	48.0 ± 1.3	49.8 ± 1.2	46.8 ± 1.2	46.5 ± 1.3	40.0 ± 1.7	<0.001
Dwelling type, apartment (%)	62.6 ± 1.2	51.5 ± 1.0	52.8 ± 1.0	56.8 ± 1.1	60.7 ± 1.5	<0.001
Occupation, blue collar (%)	18.7 ± 0.9	21.4 ± 0.8	19.7 ± 0.8	19.1 ± 0.8	16.5 ± 1.3	0.003
Stress (%)						<0.001
None	18.2 ± 0.9	12.4 ± 0.6	14.0 ± 0.5	15.9 ± 0.7	18.3 ± 1.1	
Low	43.6 ± 1.0	55.6 ± 0.9	57.5 ± 0.8	58.5 ± 0.8	54.9 ± 1.5	
High	28.3 ± 1.0	26.5 ± 0.8	24.4 ± 0.6	21.6 ± 0.8	20.7 ± 1.2	
Very High	10.0 ± 0.6	5.5 ± 0.3	4.1 ± 0.3	4.0 ± 0.3	6.1 ± 0.7	
Suicidal ideation (%)	25.9 ± 1.0	15.9 ± 0.6	13.3 ± 0.5	14.8 ± 0.6	21.8 ± 1.2	<0.001
Depression (%)	22.1 ± 0.8	13.9 ± 0.5	11.9 ± 0.5	13.1 ± 0.6	16.4 ± 1.1	<0.001
Smoking (%)						0.004
Current smoker	41.8 ± 1.0	46.6 ± 0.8	44.7 ± 0.8	45.8 ± 0.8	45.5 ± 1.5	
Ex-smoker	1.6 ± 0.2	2.0 ± 0.2	1.9 ± 0.2	1.7 ± 0.2	1.6 ± 0.2	
Never smoker	56.6 ± 1.0	51.4 ± 0.8	53.4 ± 0.8	52.5 ± 0.8	52.9 ± 1.5	
Alcohol,<1/month (%)	54.9 ± 1.1	45.2 ± 1.0	46.6 ± 0.9	48.5 ± 1.0	52.2 ± 1.5	<0.001
Allergic rhinitis (%)	9.3 ± 0.6	11.4 ± 0.5	12.3 ± 0.6	11.6 ± 0.5	10.0 ± 0.9	0.010
Chronic otitis media (%)	3.7 ± 0.4	3.6 ± 0.3	3.7 ± 0.3	3.4 ± 0.3	3.1 ± 0.5	0.842
Hypertension (%)	23.9 ± 1.0	14.7 ± 0.5	13.8 ± 0.5	13.8 ± 0.6	17.3 ± 1.0	<0.001
Angina or Myocardial infarction (%)	2.7 ± 0.3	1.7 ± 0.2	1.3 ± 0.2	1.3 ± 0.2	1.3 ± 0.2	<0.001
Diabetes mellitus (%)	9.0 ± 0.5	5.3 ± 0.3	5.3 ± 0.3	5.9 ± 0.4	7.0 ± 0.6	<0.001
Hyperlipidemia (%)	8.0 ± 0.5	6.3 ± 0.4	6.0 ± 0.4	5.3 ± 0.4	4.3 ± 0.6	<0.001
Asthma (%)	4.2 ± 0.4	2.7 ± 0.3	2.3 ± 0.2	2.3 ± 0.2	3.1 ± 0.5	<0.001
Chronic obstructive pulmonary disease (%)	1.0 ± 0.2	0.7 ± 0.1	0.7 ± 0.1	0.5 ± 0.1	0.7 ± 0.2	0.097
Bronchiectasis (%)	0.4 ± 0.1	0.3 ± 0.1	0.3 ± 0.1	0.2 ± 0.1	0.6 ± 0.2	0.264
Gastric ulcer (%)	6.5 ± 0.5	5.8 ± 0.4	4.7 ± 0.3	4.5 ± 0.3	3.7 ± 0.5	<0.001
Liver cirrhosis (%)	0.3 ± 0.1	0.1 ± 0.0	0.2 ± 0.1	0.1 ± 0.0	0.1 ± 0.0	0.008
Atopic dermatitis (%)	3.3 ± 0.4	3.0 ± 0.3	3.0 ± 0.3	3.4 ± 0.3	3.6 ± 0.6	0.740
Chronic renal failure (%)	0.6 ± 0.1	0.3 ± 0.1	0.2 ± 0.1	0.3 ± 0.1	0.8 ± 0.3	0.011

### Multivariate analysis of associations between CRS and sleep duration

The association between CRS and sleep duration after adjustment for multiple risk factors is presented in Table 3. The participants with sleep duration of 7 hours were set as the reference group for the logistic analysis. After adjusting for covariates (age, sex, household income, residency, dwelling type, education, depression, alcohol, AR, chronic otitis media, angina or myocardial infarction, asthma, chronic obstructive pulmonary disease, bronchiectasis, and gastric ulcer), a sleep duration of ≤5 hours was significantly associated with CRS (OR = 1.502; 95% CI = 1.164–1.938), compared to a sleep duration of 7 hours.

**Table 3:** Adjusted odds ratios for the prevalence of chronic rhinosinusitis according to sleep duration. Model 1: adjusted for age, sex, household income, residency, and dwelling type. Model 2: adjusted for age, sex, household income, residency, dwelling type, education, depression, and alcohol use. Model 3: adjusted for age, sex, household income, residency, dwelling type, education, depression, alcohol use, allergic rhinitis, chronic otitis media, angina or myocardial infarction, asthma, chronic obstructive pulmonary disease, bronchiectasis, and gastric ulcer.

		≤5 hours/day	6 hours/day	7 hours/day	8 hours/day	≥9 hours/day
		OR (95% CI)	OR (95% CI)	OR (95% CI)	OR (95% CI)	OR (95% CI)
Total	Model 1	1.532 (1.187–1.978)*	1.053 (0.852–1.301)	1	0.856 (0.679–1.079)	1.030 (0.738–1.437)
	Model 2	1.511 (1.169–1.952)*	1.054 (0.853–1.302)	1	0.858 (0.680–1.082)	1.031 (0.738–1.442)
	Model 3	1.502 (1.164–1.938)*	1.046 (0.848–1.290)	1	0.865 (0.685–1.091)	1.051 (0.750–1.472)
Age <50	Model 1	1.386 (0.956–2.009)	1.018 (0.779–1.331)	1	0.818 (0.617–1.085)	0.880 (0.576–1.343)
	Model 2	1.363 (0.941–1.974)	1.017 (0.779–1.328)	1	0.822 (0.619–1.090)	0.887 (0.578–1.360)
	Model 3	1.363 (0.939–1.979)	1.011 (0.776–1.318)	1	0.822 (0.619–1.091)	0.882 (0.575–1.352)
Age ≥50	Model 1	1.603 (1.130–2.274)*	1.146 (0.814–1.613)	1	0.905 (0.616–1.330)	1.168 (0.712–1.917)
	Model 2	1.608 (1.133–2.283)*	1.146 (0.814–1.614)	1	0.905 (0.616–1.331)	1.186 (0.722–1.937)
	Model 3	1.602 (1.123–2.286)*	1.153 (0.816–1.631)	1	0.936 (0.634–1.384)	1.268 (0.766–2.101)

*Significant at *p* < 0.05.

### Effect of age on association between CRS and sleep duration

For the subgroup analysis, age was divided into younger (20–49 years old) and older (≥50 years old) subgroups. The prevalence of CRS was 4.6% in younger subgroup (20–49 years old) and 3.9% in older subgroup (≥50 years old) (*p* = 0.009). In the logistic analysis, CRS was significantly associated with sleep duration only in older subgroup. All of the adjusted models showed that, in the older subgroup, the sleep duration ≤5 hours showed higher odds for CRS in reference to sleep duration of 7 hours (OR = 1.602; 95% CI = 1.123–2.286) (Table 3). However, the association between CRS and sleep duration was not significant in younger subgroup.

## Discussion

Several epidemiologic studies have documented that short and/or long sleep duration is associated with adverse health outcomes^5–10^. However, little is known about the specific relationship between sleep duration and CRS. To our knowledge, the present study is the first to evaluate the association between sleep duration and CRS, based on general population data. A potential strength of this study is the large number of subjects enrolled. Our study showed that self-reported sleep duration was significantly associated with CRS. When we analyzed the effect of age, significantly shorter sleep duration in subjects with CRS was observed only in the adult group aged 50 years and older. However, there was no significant difference of sleep duration in younger adults aged between 20 and 49 years. It is assumed that sleep of older adults might be more impaired by the presence of CRS. Given that sleep duration is known to decrease with age^14^, older people might perceive more strongly that their sleep was impaired by factors that can disturb their quality of sleep, and report short sleep time. In some diseases such as obesity, there was a study suggesting age dependency between sleep duration and obesity^15^. There also have been studies that reported age-dependent associations between sleep duration and diseases such as metabolic syndrome^10^ and hypertension^16^. However, studies showing age dependency between sleep duration and CRS are lacking.

Several studies have demonstrated that patients with CRS have reduced sleep quality and poor quality of life^17–19^. A systematic review suggested that bothersome symptoms of rhinosinusitis is associated with sleep disordered breathing and is thought to be a key cause of sleep impairment^19^. However, recent population-based cohort study^20^ showed that patients with obstructive sleep apnea (OSA) had a higher risk of CRS compared to patients without OSA. They explained that abnormal inflammatory reactions of OSA patients might contribute to the development and exacerbation of CRS. Another study also reported that chronic inflammation and intermittent hypoxia due to OSA can cause inflammatory cytokine release and contribute to mucosal and muscular inflammation of the upper airway^21^. Nonetheless, the possibility that short sleep duration might influence the development of CRS has not previously been investigated.

The mechanism mediating the association between sleep duration and chronic diseases is unclear but systemic inflammation or oxidative stress has been reported to be possible mechanisms^22,23^. Patients with CRS demonstrated a reduced antioxidative tissue status^24^. Although we could not explore the mechanism because of a limitation as a cross-sectional study, short sleep duration might induce oxidative stress and be involved in the development of CRS or systematic inflammation. The biggest limitation of KNHANES is that this cohort includes only cross-sectional data. Therefore, the limitation of our study is also just an association study using this cross-sectional data. So, it is not possible to identify a causal relationship between reduced sleep duration and CRS and we could only present possible mechanisms.

Our study also has limitations. As of now, KNHANES does not include any standardized sleep assessment questionnaires, such as Epworth sleepiness scale, Pittsburg Sleep questionnaire, and Berlin questionnaire. Sleep duration could not also be measured objectively using actigraphy or polysomnograpy because it was a nationwide survey study. However, self-reporting may be considered valuable in a large-scale epidemiologic study. Additionally, a previous study has reported a good correlation between self-reported and objectively measured sleep duration^25^. Moreover, self-reported sleep duration is considered to be more accurate in detecting long-term sleep habits and more suitable for epidemiologic studies^23^. Because the present study was an association study using cross-sectional data, it was not possible to identify a causal relationship between self-reported sleep duration and CRS. In future, KNHANES should develop a prospective sampling strategy. That is, some of the KHNANES cohort should be prospectively sampled and followed up consecutively to identify causal relationships. Diagnosis of CRS was estimated based on a questionnaire and there is a possibility of bias associated with self-reported diseases. However, questionnaires are widely accepted for epidemiologic studies and questions included the diagnosis made by physicians. Certain environmental factors may contribute to the comorbidities and covariables because some of participants will share the same household. Even though we could not completely exclude the influence of environmental factors associated with the same household such as living circumstances and dietary habits, we tried to minimize these effects using logistic regression models including household income, residency, and dwelling type.

In conclusion, our study suggests that shorter sleep duration may be negatively associated with CRS in older Korean adults. This study is the first to focus on the association between sleep duration and CRS in large population-based examination survey collected over 5 years. Further investigations are needed to elucidate the underlying mechanisms of the relationship between shorter sleep duration and CRS.

## Methods

### Study population and data collection

We used data from the KNHANES III and IV, which was conducted between 2005 and 2007–2009. The KNHANES III (2005) was conducted by the Korea Institute for Health and Social Affairs (KIHASA), the Korea Health Industry Development Institute (KHIDI), and the Korea Centers for Disease Control and Prevention (KCDC). The KNHANES IV (2007–2009) was conducted solely by the KCDC. A total of 65,850 individuals of 25,300 households were asked to participate in this survey. The survey was carried out using a complex, stratified, multistage probability sample design. The sample represents the non-institutionalized civilian population of South Korea. Among the 65,850 individuals selected, 57,480 (87.3%) agreed to participate in this survey. This study enrolled 43,371 participants aged ≥20 years. Analyses were conducted on data from 24,658 subjects who answered the sleep duration questionnaire. The KCDC obtained written and informed consent from all participants. All study protocols were approved by KCDC Institutional Review Board (Approval no. 2007-02CON-04-P, 2008-04EXP-01-C, and 2009-01CON-03-2C). For users and researchers who promise to follow the research ethics throughout the world, micro-data (in the form of SAS and SPSS files) can be downloaded by e-mail and the details of KNHANES can be accessed on the KNHANES website in English version (http://knhanes.cdc.go.kr/knhanes/eng/index.do).

### Sociodemographic characteristics

All subjects underwent interviews conducted by trained interviewers of the KCDC. A set of structured questions was asked. All data were converted to appropriate categories for analysis. Age, education, household income, residency, and occupation were categorized as previously described^26^. The type of dwelling was categorized into apartments and detached houses. Stress level was categorized into four subgroups: none, low, high, and very high. Suicidal ideation was classified into either “yes” or “no” and was extracted from responses to the question “Have you wanted to die during the last year?” Smoking status was categorized as never smoker (persons who have never smoked), ex-smoker (persons who smoked in the past but do not smoke presently), and current smoker (persons who smoke presently). Alcohol use was obtained through questioning the subjects about their average frequency (per week or month) of alcohol consumption during the last year.

### Definition of chronic rhinosinusitis, medical disease, and sleep duration

Participants were considered to have physician-diagnosed CRS if they responded positively to “Have you been diagnosed with CRS by a physician?” Other chronic diseases (AR, chronic otitis media, hypertension, angina or myocardial infarction, diabetes mellitus, hyperlipidemia, asthma, chronic obstructive pulmonary disease, bronchiectasis, gastric ulcer, liver cirrhosis, atopic dermatitis, and chronic renal failure) were also defined as a self-reported physician’s diagnosis. Sleep duration was determined from the self-reported questionnaire response to “How much on average do you sleep each day?”

### Statistical analysis

The sampling weight was constructed for sample participants to represent the non-institutionalized civilian Korean population by the complex sample design, survey non-response, and post-stratification. The weight based on the reciprocal of the selection probabilities (psu, household), inverse of response rate (household, subject), and a post-stratification factor to make the resulting survey estimates for age- and sex-specific Korean population. The survey sample weights were used in all analyses. Data are presented as the mean ± standard error (SE) for continuous variables or as percentage ± SE for categorical variables. The subjects were divided into 5 subgroups according to sleep duration: ≤5 hours, 6 hours, 7 hours, 8 hours and ≥9 hours. The proportion of categorical variables was compared by Pearson’s chi-square (χ^2^) tests. The associations between CRS and various sociodemographic categories and medical diseases were explored. The prevalence of CRS and the characteristics of the study population according to sleep duration were measured. In addition, the logistic regression analysis was used to study the relationship between CRS and sleep duration. Three models were constructed: in model 1, adjustments were made for age, sex, household income, residency, and dwelling type; model 2 included additional adjustments for education, depression, and alcohol use; and in model 3, adjustments for AR, chronic otitis media, angina or myocardial infarction, asthma, chronic obstructive pulmonary disease, bronchiectasis, and gastric ulcer were added. Results are presented as odds ratios and 95% confidence interval. A *p*-value < 0.05 was considered significant. Missing data were considered to be missing completely at random. The data were analyzed using SPSS (complex version 18.0, SPSS Inc., Chicago, IL, USA).

## Data Availability

All the data generated and/or analyzed during the current study are included in this article and are available from the corresponding author on reasonable request.
